# When Air Is Not an Emergency: Using Intraperitoneal Sevoflurane to Rule Out Perforation

**DOI:** 10.7759/cureus.107899

**Published:** 2026-04-28

**Authors:** Riddhi Machchhar, Matthew Friedland, Tejaswi Makkapati, Eric Ball, Neil Patel, Emad Kamel, Stephen Klepner

**Affiliations:** 1 Internal Medicine, Ocean University Medical Center, Hackensack Meridian Health, Brick Township, USA; 2 Dermatology, Yale School of Medicine, New Haven, USA; 3 Physical Medicine and Rehabilitation, John F. Kennedy (JFK) University Medical Center, Hackensack Meridian Health, Edison, USA; 4 Pulmonary and Critical Care Medicine, Ocean University Medical Center, Hackensack Meridian Health, Brick Township, USA; 5 General Surgery, Ocean University Medical Center, Hackensack Meridian Health, Brick Township, USA

**Keywords:** asymptomatic pneumomediastinum, atraumatic, bleb, idiopathic spontaneous pneumoperitoneum, pulmonary bleb, sevoflurane

## Abstract

Idiopathic pneumoperitoneum (IP) is a rare diagnosis of exclusion typically discovered during imaging for unrelated concerns. Most pneumoperitoneum cases are secondary to visceral perforation, necessitating urgent surgical intervention. However, IP is a distinct, non-surgical condition that can present asymptomatically, complicating clinical decision-making. A 76-year-old female with multiple comorbidities presented to the emergency department after her Holter monitor detected intermittent, third-degree heart block. She was asymptomatic from a gastrointestinal standpoint, with a benign abdominal exam and normal inflammatory markers. However, chest imaging incidentally revealed pneumomediastinum and pneumoperitoneum, raising concern for gastrointestinal perforation. A diagnostic laparotomy was performed after temporary transvenous pacing was established. No visceral perforation was identified. Interestingly, sevoflurane administered through an endotracheal tube was detected in the abdominal cavity, suggesting a thoracoabdominal communication, possibly from a ruptured mediastinal bleb. She remained hemodynamically stable throughout hospitalization, had a permanent pacemaker placed, and was discharged in stable condition. This case highlights an unusual presentation of asymptomatic idiopathic pneumoperitoneum with suspected thoracoabdominal gas tracking, confirmed by intraoperative detection of sevoflurane. It underscores the importance of cautious interpretation of incidental pneumoperitoneum findings and the need to consider conservative management in the absence of peritonitis.

## Introduction

Idiopathic pneumoperitoneum (IP) is a rare condition in which air is found in the abdominal cavity without a clear cause and is diagnosed after other causes are excluded [1-3]. Most cases of pneumoperitoneum are due to a hole in the gastrointestinal (GI) tract and require emergency surgery, making up about 90% of cases [1,2,4]. Other findings of free air include pneumatosis intestinalis, in which air is trapped within the bowel wall, and portal venous gas, in which air is present within the portal vein. Both conditions are often a result of acute bowel ischemia and are associated with mortality rates ranging from 75-90% [2,5-7]. Some cases of pneumoperitoneum occur without bowel perforation and are referred to as spontaneous or non-surgical pneumoperitoneum. These are often the result of abdominal, chest, gynecologic, or other medical procedures and are treated conservatively [8]. When no alternative cause is found, IP is diagnosed, and patients are usually managed conservatively with close observation [8]. We present a case utilizing sevoflurane to assess for perforation in the setting of diagnosed spontaneous pneumoperitoneum and pneumomediastinum.

## Case presentation

A 76-year-old female with a past medical history of type 2 diabetes mellitus (hemoglobin A1c: 6.5%) with complications of Charcot foot and left third toe amputation, hyperlipidemia, medullary thyroid cancer status post thyroidectomy, congestive heart failure with a recovered ejection fraction, obsessive compulsive disorder, and hoarding disorder presented to the emergency room after her outpatient Holter monitor showed intermittent, third-degree heart block. She denied any symptoms or sensations from the arrhythmia.

On initial presentation in the emergency room, she was afebrile with blood pressure of 135/78 mmHg, heart rate mostly in 60s (beats per minute) with occasional sustained dips to 40s (beats per minute), respiratory rate 16 breaths per minute, and oxygen saturation of 95% on room air. Labwork was notable for normal white blood cell, red blood cell, and platelet count. A comprehensive metabolic panel showed sodium 134 mEq/L, blood urea nitrogen (BUN) 29 mg/dL, and creatinine 1.11 mg/dL; the remaining values were within normal limits. Magnesium, phosphorus, lactic acid, and brain natriuretic peptide (BNP) were normal. The electrocardiogram showed third-degree heart block with a ventricular rate of 38 bpm. Cardiology was consulted. The first sign of unsuspected abnormality was noted on the emergency room chest radiograph.

**Figure 1:**
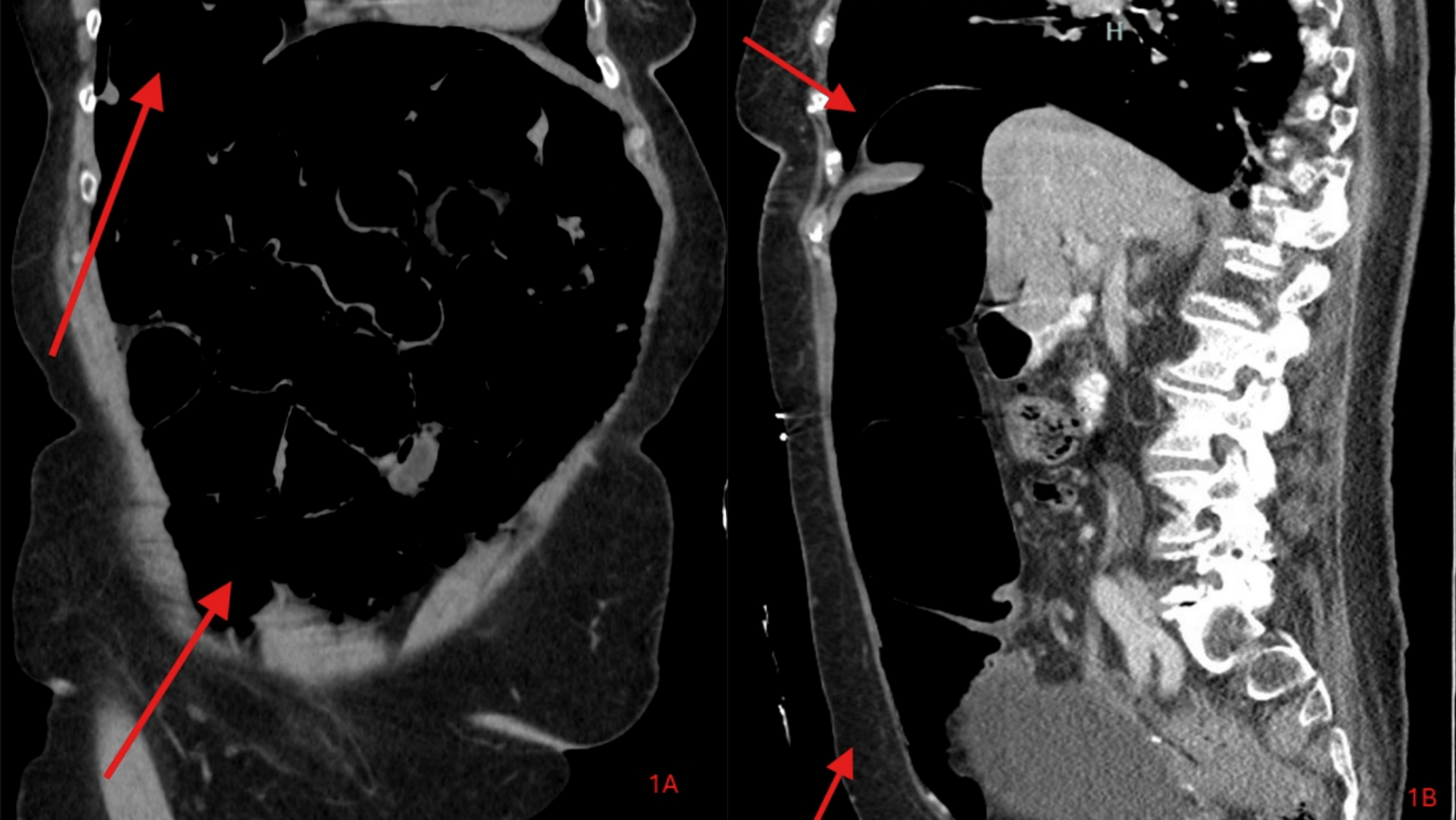
Computed tomography of abdomen and pelvis (A, B). Pneumomediastinum and pneumoperitoneum, concerning for possible colonic perforation, though no clear area of perforation was noted on imaging. Arrows represent air found in the mediastinum and peritoneum.

**Figure 2:**
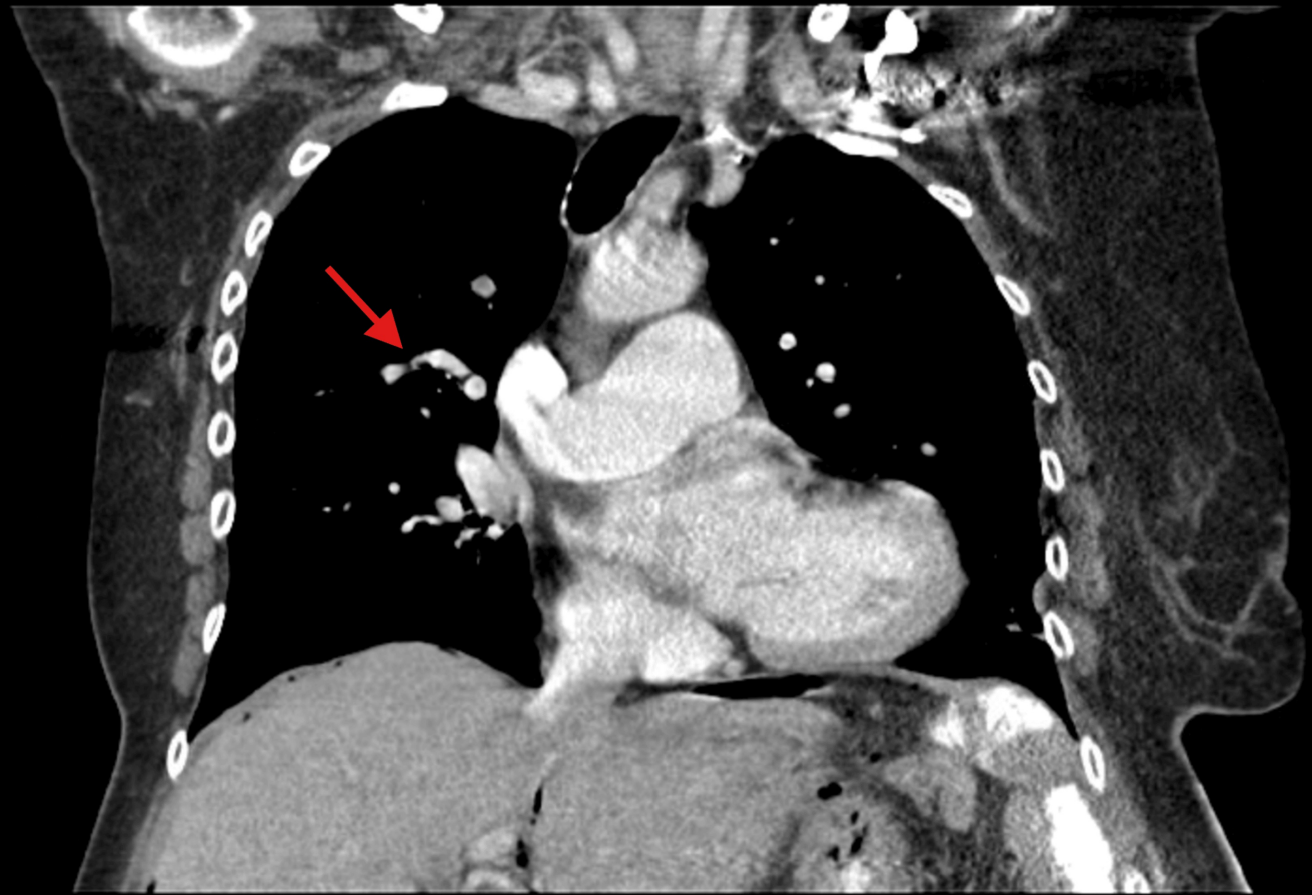
Pulmonary embolism identified on admission CT chest/abdomen/pelvis (CT C/A/P); the arrow indicates a filling defect.

The patient was accepted to the intensive care unit (ICU) for monitoring with a plan to place a permanent pacemaker. Due to concerns for pneumomediastinum on initial chest X-ray, cardiothoracic and general surgery were consulted, and a computed tomography (CT) of the chest, abdomen, and pelvis was obtained. The CT showed a bilateral pulmonary embolism, pneumomediastinum, and pneumoperitoneum, concerning for possible colonic perforation, though no clear area of perforation was noted on imaging (Figures 1A, 1B, 2).

She remained hemodynamically stable with active bowel sounds and a soft, non-tender, non-distended abdomen, without rigidity, rebound, or guarding. Labwork remained within normal limits. In preparation for diagnostic laparotomy, a temporary transvenous pacemaker was placed for hemodynamic support during surgery until a permanent pacemaker could be placed. General surgery performed a diagnostic laparotomy, during which no perforation was seen. During the procedure, the smell of sevoflurane in the abdomen alerted the surgical team to a possible communication between the thorax and abdomen, such as a burst mediastinal bleb. After the operation, the abdominal free air remained, but now the clinical concern for a perforation of the viscus or colon was very low. The patient was discharged in stable condition following a successful pacemaker placement with plans for close outpatient follow-up and monitoring. She was also started on oral anticoagulation for pulmonary embolism discovered on CT.

## Discussion

Idiopathic pneumoperitoneum (IP) is a rare diagnosis of exclusion. While 90% of free air cases stem from emergency GI perforations, IP and pneumatosis intestinalis represent non-surgical or ischemic variants. Both of these manifestations can be symptomatic and have a mortality rate of 75-90% [2,7]. A spontaneous or “non-surgical” pneumoperitoneum, which may arise from abdominal, thoracic, gynecological, or iatrogenic causes, can be managed conservatively [8]. When the etiology of free air in the peritoneal cavity is unknown, IP becomes a working diagnosis and usually entails conservative management with serial monitoring [8]. Interestingly, only a handful of IPs have been incidentally found in asymptomatic patients with an uncomplicated clinical history and benign course [9].

Even with a known etiology, free air in the peritoneal cavity has a few theories regarding its pathophysiology [10]. One is the mechanical theory postulating that increased pressure in the lungs, mediastinum, or bowel lumen can lead gas to enter the bowel wall's serosal and/or mucosal lining [10,11]. Free air in the pneumoperitoneum could also be due to intramural gas blebs fissuring, leading to free air in the serosal and mucosal layers of the bowel, which was a working etiology in our patient [10,12]. Of significance, the submucosal and subserosal layers are not directly associated with the digestive process as they are separated from the lumen of the bowel wall, which maintains the integrity of the GI tract [13]. As seen in our patient, it is noted that tracking of air from the mediastinum into the retroperitoneum and peritoneal cavity is possible. However, unlike the reported cases, our patient did not experience barotrauma, pneumothorax, cardiopulmonary resuscitation, or invasive ventilation.

As there are limited case reports of asymptomatic IP, one such report evaluated the known cases and available literature to create an algorithm for pneumoperitoneum management and IP recognition [1]. As of 2021, nine asymptomatic IP cases have been documented with a near-even distribution between genders spanning an age range of 52-85 years and a mean age of 72.9 years [1]. While two patients had respiratory symptoms, the remaining IP cases were detected during health screenings using chest radiography (CXR) and abdominal computed tomography (CT) [1,3]. All patients were managed conservatively, with 25% experiencing recurring IP at three and five years later [1].

Despite our patient having a benign abdominal exam and no signs and symptoms of peritonitis, she was taken to the OR for a diagnostic exploratory laparotomy after a multispecialty team discussion was held in order to definitively rule out perforation. IP can be considered a more incidental imaging finding rather than a disease diagnosis, as there are no associated chief complaints relative to IP [14]. In fact, with the increased utilization and quality of our imaging modalities, an increased appreciation of idiopathic pneumoperitoneum may occur [1].

Currently, there are no standardized guidelines for the management of asymptomatic idiopathic spontaneous pneumoperitoneum [15]. Of the few reported cases of IP, diagnostic laparotomies have been done to assess for intestinal perforation [16]. Although imaging revealed no clear perforation explaining the pneumomediastinum and pneumoperitoneum, the detection of sevoflurane in the abdomen while undergoing the exploratory laparotomy with an intubated patient confirmed the presence of an abnormal connection between the thorax and abdomen. The detection of sevoflurane was an important finding because, although the diagnostic laparotomy was unremarkable, this finding further supported that the pneumoperitoneum was not secondary to a GI perforation.

With the diagnostic laparotomy negative, other causes of pneumoperitoneum must be considered, such as esophageal perforation or ruptured mediastinal bleb [17]. Although esophageal perforation could explain the observed pneumomediastinum and pneumoperitoneum, it was not suspected in this patient due to the absence of classic symptoms, such as chest pain, fever, or dysphagia; therefore, further imaging was not pursued.

Although diagnostic laparotomy is generally not indicated for asymptomatic pneumoperitoneum, our patient’s advanced age, comorbidities, and presentation of third-degree heart block made close monitoring alone unreliable due to fear of masked peritonitis, leading to the decision to operate.

## Conclusions

Our case presents a rare occurrence of asymptomatic idiopathic pneumoperitoneum with a benign abdominal exam, discovered incidentally in the evaluation of cardiac conduction abnormalities. The presence of sevoflurane in the peritoneal cavity, despite no evidence of gastrointestinal perforation, suggests translocation of anesthetic gas across an abnormal thoracoabdominal communication, such as a ruptured mediastinal bleb. Our experience with this case suggests that, in hemodynamically stable, asymptomatic patients, a more conservative approach might be considered to avoid unnecessary surgical exploration, provided that close monitoring is maintained. As imaging modalities improve and become widely used, incidental findings such as IP may become more common, reinforcing the need for standardized guidelines and algorithms for diagnosis and management.
